# Risk factors for developing jumper's knee in sport and occupation: a review

**DOI:** 10.1186/1756-0500-2-127

**Published:** 2009-07-08

**Authors:** Ivo JH Tiemessen, P Paul FM Kuijer, Carel TJ Hulshof, Monique HW Frings-Dresen

**Affiliations:** 1 Coronel Institute of Occupational Health, Academic Medical Center (AMC), University of Amsterdam, PO Box 22700, 1100 DE Amsterdam, The Netherlands

## Abstract

**Background:**

The onset of jumper's knee is generally associated with sports and sporting activities. Employees in certain professions might be at risk as well for developing jumper's knee. Therefore, it is of interest to identify risk factors in sport and/or occupation.

**Findings:**

A systematic search of the international scientific literature was performed until November 2008 in the scientific databases (a) Medline, (b) Embase, and (c) SportDiscus. All types of studies were included. The search strategy retrieved ten articles about risk factors in sport that met the inclusion criteria. Risk factors that could be identified are; playing volleyball (4 studies), playing basketball (3 studies), training and playing volleyball/basketball more than 12 hours per week (2 studies), in combination with weight-bearing activities of at least 5 hours per week (1 study) and playing or training on a hard surface (1 study). No studies were found regarding occupation that fulfilled the inclusion criteria.

**Conclusion:**

Playing volleyball and basketball has a positive association with the onset or worsening of jumper's knee. Other risk factors are training and playing hours of at least 12 hours per week and/or in combination with weight training of at least 5 hours per week, and/or with playing or training on a hard surface. We did not find a specific occupational risk factor.

## Background

Jumper's knee is among the most frequent injuries in sports [1,2] and it has upset many professional sporting careers. The prevalence of jumper's knee ranged from 30%–51% in volleyball [3-10]; 25%–32% in basketball [2,8]; to 0% for cycling and wrestling [2]. The term "jumper's knee" was first introduced by Blazina et al. [11]: a gradual insidious onset of aching in the knee centered over the infrapatellar or suprapatellar region, especially localized to the superior or inferior poles of the patella. It is generally accepted that jumper's knee is caused by a dynamic overload, mainly eccentric, of the extensor mechanism of the knee joint [12-14]. Jumper's knee is associated with sports and sporting activities, especially with repetitive activities such as jumping, climbing, kicking or running [12-14]. Therefore, jumper's knee is perhaps a misleading term.

Not only with sports and sporting activities, but also in certain professions, like policemen or fire-fighters, people might be at risk for developing jumper's knee. Kinsella [15] wrote that the vast majority of semi-professional athletes performing a variety of occupations complain of symptoms similar to jumper's knee as a result of work-related activities, including walking up and down stairs or sitting for extended periods. If certain professions are indeed at stake, the high rate of recurrence [14] and the risk of the condition becoming chronic might result in work-related complaints, decreased work ability and increased sick leave. It is currently unknown, however, if jumper's knee can be empirically related to work and working activities.

The aim of this study is to systematically search the literature to find evidence for risk factors that are associated with the development of jumper's knee in sport and/or occupation.

## Methods

### Search strategy

We systematically searched the literature until 19 November, 2008 in (a) Medline (biomedical literature) (b) Embase (1980–2008), biomedical and pharmacological literature and (c) SportDiscus (EBSCOhost, including CINAHL plus). The first step in the search strategy was the combination of the search terms for jumper's knee or its synonyms (such as jumper's knee, patellar tendinopathy OR patellar tendonitis OR patellar tendonitis OR patellar insertion tendinopathy OR chronic patellar tendinopathy OR infrapatellar tendonitis OR insertion tendonitis OR tendinopathy OR quadriceps tendinopathy OR quadriceps tendonitis OR quadriceps tendonitis, all combined with knee), along with specific search terms for risk factors in sport and/or occupation. The specific search strategy and the inclusion criteria are described in a report [16].

Step 2 consisted of the application of the general inclusion criteria to title and abstract. Step 3 applied the specific inclusion criteria, mainly concerning the description of exposure to specific tasks or activities, to the full text of the article after exclusion of the duplicates. These specific inclusion criteria were defined to ensure capturing all relevant studies. Step 4 was defined as the "snowball method." This consisted of three actions: (1) we checked the references of the included full text articles; (2) we used the option 'related articles' in PubMed and (3) we performed a forward search with 'web of science' for highly relevant full-text articles. The remaining articles were the outcome of our systematic search strategy. Application of the general and specific inclusion criteria was performed by the primary researcher (IT); for points requiring clarification, the second researcher (PK) was consulted. Decisions were made based on consensus.

### Data extraction

The primary researcher (IT), after reading the full text of the included articles, selected the relevant information to include in table 1. The publication date, the country, the study design were considered relevant information, as well as whether the research was performed in a sports or an occupational setting. Second, all relevant data concerning the research question was included. The second (PK) and fourth researcher (MF) read the extracted data and made additions or revisions.

**Table 1:** Included studies regarding risk factors in sport and/or occupation described in terms of author/year/country, sport/occupation, design of the study (cross sectional, case-control or cohort), population, exposure and results.

**1e author/year/country**	**Sport/Occupation**	**Research design**	**Population**	**Exposure**	**Results**
Cook/2000a/ Australia	Sport	Cohort prospective (16 months)	N = 26, 8 males, 18 females, A = [14–18] All a-symptomatic at baseline Activity: elite basketball players	-sport hrs/week: unknown, -14.5 hrs of weight-bearing training per week	-significant increase in tendon abnormalities was associated with significant increase in training hrs/week

Cook/2000b/ Australia	Sport	Cross- sectional	N = 163, two groups: -N = 134, elite basketball players, 70 males 64 females, A = 16 [14–18], H = 186 [162–211], W = 75 [47–98] -N = 29, swimming athletes acting as controls, 17 males, 12 females, A = 17 [31–21], H = 174 [154–192], W = 62 [38–82]	-sport hrs/week basketball players: 15 hrs exercise, 12 hrs weight bearing -sport hrs/week swimmers: 17 hrs exercise, 3 hrs weight bearing	-at least 7 percent of the basketball players had jumper's knee but none of the swimmers

Crossley/2007/Australia	Sport	Case control	N = 58, three groups; -no symptoms, N = 31, A = 24 ± 6, H = 177 ± .0.9, W = 71 ± 11 -unilateral JK, N = 14, A = 26 ± 7, H = 178 ± 1, W = 80 ± 16 -bilateral JK, N = 13, A = 28 ± 8, H = 176 ± 1, W = 82 ± 14 Activity: tennis, volleyball, basketball, netball or soccer	Sport hrs/week: - no symptoms group: 3 hrs - unilateral group: 4 hrs - bilateral group: 7 hrs	-significantly more hrs of sport per week in bilateral group compared to both unilateral and no symptoms group -no significant difference in hrs of sport per week in unilateral group compared to the no symptoms group

Ferretti/1984/ Italy	Sport	Cross- sectional	N = 407, both males and females Activity: elite volleyball players	Sport hrs/week: -between 2 and more than 14 hrs	- the number of playing and training sessions (> 14 hrs/week) per week increased the prevalence of jumper's knee -years of play had no significant effect, but peak is seen at third year of participation -playing surface (p < 0.05) (parquet better than cement) -type of training: no effect

Gaida/2004/ Australia	Sport	Case control	N = 39, all elite female basketball players, three groups; -no JK (controls), N = 24, A = 21 ± 3, H = 176 ± 7, W = 74 ± 9 -unilateral JK, N = 8, A = 20 ± 2, H = 178 ± 10, W = 73 ± 13 -bilateral, N = 7, A = 21 ± 3, H = 178 ± 9, W = 74 ± 9 Activity: basketball players	Sport hrs/week: -Unilateral and bilateral group on average: 12 hrs -controls on average: 9 hrs	-subjects with 1 or 2 hypoechoic regions trained 2.6 (± 1.4) hrs/week more than controls

Kettunen/2002/Finland	Sport	Case control Prospective (15 years)	N = 47, (all males) two groups: -N = 14, no JK (controls) -N = 18, with JK (cases) Activity: ball players, long distance runners	Sport hrs/week: Not mentioned but based on table 2: about 10	-no difference in duration, frequency and intensity of work and leisure time physical activity between two groups at follow up

Lian/2003/ Norway	Sport	Case control	N = 47, all males two groups: -N = 24 with JK (cases), A:22 ± 4 H = 191 ± 7, W = 87 ± 8 -N = 23 no JK (controls), A = 22 ± 4, H = 190, W = 82 ± 8 Activity: volleyball	Sport hrs/week: cases: 8 hrs, controls:7 hrs Weight training hrs/week: cases: 5 hrs, controls: 2 hrs	-no association between hours of training and JK (p > 0.05) -more weight training is associated with JK (p < 0.01)

Malliaras/ 2006/ Australia	Sport	Cross sectional	N = 113, 73 males and 40 females, A:26 ± 5, H = 79 ± 13, W = 181 ± 0 activity: volleyball	-years of exposure: 4.6 ± 1.6 -sport hrs/week: 8.4 ± 4.6	-no association between the years of volleyball playing and the weekly hours of training with tendon abnormality and/or pain

Taunton/2002/Canada	Sport	Cohort prospective (two years)	Patellar tendinopathy (JK) N = 96, A:34, 55 males, H = 171, W = 83 41 females, H = 159, W = 64 Activity: Running	-years of exposure: 10.0 ± 3.7 -sport hrs/week: 6.1 ± 0.7	-no association between hours of training and JK (p > 0.05)

Warden/2007/USA (Indianapolis)	Sport	Case control	N = 63, two groups; -symptomatic, 30: 20 males, 10 females, A = 27 ± 7, H = 177 ± 1, W = 80 ± 16 - a-symptomatic, 33: 22 males, 11 females, A = 25 ± 7, H = 177 ± 1, W = 72 ± 12	Sport hrs/wk: Symptomatic group: 4.2 ± 2.7 A-symptomatic group: 3.4 ± 1.6	-no significant difference in sport hours per week between symptomatic and a symptomatic group

N = number of subjects, hrs = hours, wk = week

A = age (yrs) ± Sd or [] = range, H = height (cm) ± Sd, W = weight (kg) ± Sd,

hypoechoic region = fluid regions

## Results

### Risk factors in sport and/or occupation

#### Retrieved studies

**Figure 1:**
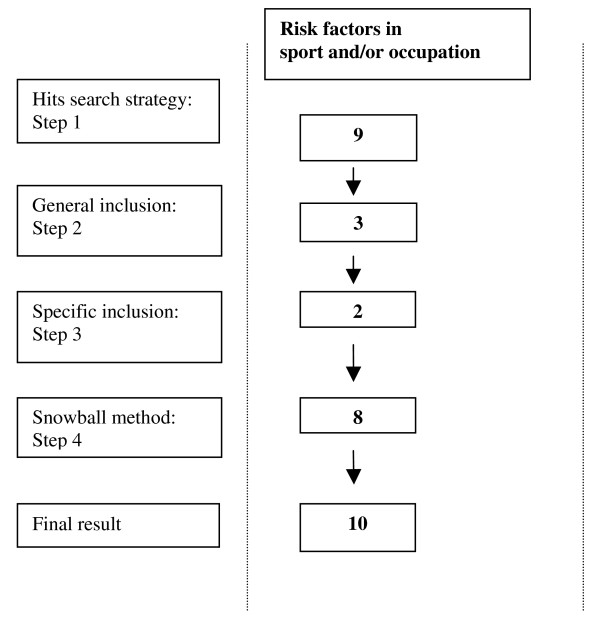
**Flow chart of the search strategy (consisting of 4 steps) and the corresponding hits from the databases (Medline, Sportdiscus and Embase) tailored for risk factors in sport and/or occupation**.

The results of the systematic literature search for the risk factors in sport and/or occupation are presented in figure 1. In total, ten articles met our inclusion criteria.

#### Risk factors

All ten retrieved studies were performed in a sports setting. We found no studies reporting on jumper's knee in an occupational setting (table 1). The case-control study of Lian et al. [7] compared 24 amateur volleyball players in the top division of the Norwegian competition with current jumper's knee with 23 of their counterparts without a history of jumper's knee. The cases significantly performed more hours of weight training per week (cases: 5 hours per week versus controls: 2 hours per week). The cases did not differ from the controls regarding the hours of jump training per week (cases: 0.4 hours per week versus controls: 0.6 hours per week) or the hours of volleyball training per week (cases: 8 hours per week versus controls: 7 hours per week). No association between exposure time and symptoms of jumper's knee was found.

Warden et al. [17] performed a case-control study including 30 cases with jumper's knee and 33 activity-matched controls without jumper's knee. The hours of sport activities per week, mainly volleyball, basketball and soccer, were not significantly different between the two groups: 4 hours per week versus 3 hours per week. This result is in agreement with the results of the case-control study of Kettunen et al. [18]. In this study, the 18 athletes with jumper's knee (mostly ball players and long-distance runners) and 14 control athletes without jumper's knee (also mostly ball players and long-distance runners) did not differ in the hours of physical activity per week. Notably, no mean hours of physical activity per week were reported. However, based on our calculations from table 1 in their study, the average number of hours was approximately ten. Taunton et al. [19] conducted a retrospective cohort study of two years among a subgroup of 96 runners with jumper's knee and found that the average number of hours per week of training (6) was not associated with an increased risk. Malliaras et al. [20] investigated, in a cross-sectional study, the association between years of volleyball playing and the weekly hours of training and playing over a period of 7 months, among male and female players in the Victorian State League competition in Australia. The mean number of years of volleyball playing was eight and the mean weekly hours of training and playing was five. The years of volleyball playing and the weekly hours of training had no relation with tendon abnormality and/or pain.

Crossley et al. [21] compared in their case-control study the number of sporting hours per week among three groups: 31 controls with no jumper's knee, 14 cases with jumper's knee in one leg and 13 cases with jumper's knee in two legs. They found mixed results regarding the reported number of sport hours per week. The number of sport hours in the cases with jumper's knee in both legs was significantly higher than in the cases with jumper's knee in one leg as compared to controls: 7 hours a week versus 4 hours and 3 hours per week, respectively (mean difference: 3.2 hours per week, 95% CI 0.6–5.8). The latter two groups did not differ in sport hours per week.

However, other studies did find an association between hours of training and jumper's knee. A cross-sectional study by Ferretti et al. [10] on elite-volleyball players (>14 hours per week) concluded that the number of playing and training sessions per week increased the prevalence of jumper's knee: 3% with two sessions a week, 15% with 3 sessions, 29% with 4 sessions and 42% with >4 sessions. Cook et al. [22] performed a prospective cohort study over a period of 16 months among 26 elite junior basketball players (number of playing and training hours is not mentioned; 14.5 hours of weight bearing activities per week). They found that 30% of the basketball players with hypoechoic tendons and 7% of the basketball players without hypoechoic tendons developed jumper's knee. Moreover, the significant increase in training volume for men was associated with a significant increase in tendon abnormalities. Cook et al. [23] performed a cross-sectional study among elite junior basketball players (n = 134) and state-level swimmers (n = 29). They documented 15 hours of exercise per week and 12 hours of weight-bearing exercise per week for the basketball players. For the swimmers, they reported 17 hours of exercise per week and 3 hours of weight-bearing exercise. At least 7% of the basketball players had jumper's knee but none of the swimmers had the condition. Gaida et al. [24] compared, in a case-control study, the number of training hours per week among elite female basketball players with no hypoechoic tendons (controls, n = 24), hypoechoic tendon in one leg (unilateral cases, n = 8) or in both legs (bilateral cases, n = 7). They found that in the preceding one to six months, both types of cases trained about 3 hours per week more than the controls. The cases trained on average 12 hours per week and the controls trained 9 hours per week on average.

Finally, Ferretti et al. [10] found a statistically significant association between a hard playing surface (cement versus parquet) and an increased prevalence of jumper's knee.

## Discussion

We identified risk factors for developing jumper's knee in sports but we were not able to identify risk factors in occupation due to a lack of studies. For the elite sports of professional volleyball and basketball an association between the prevalence of jumper's knee on one hand and, on the other, training and playing hours of at least 12 hours per week and/or weight training of at least 5 hours per week and/or playing or training on a hard surface exists. Although no studies were found on an association between jumper's knee and occupation, we believe that our search strategy was sufficient: we used the most sensitive search string for "work" from the Cochrane Occupational Health Field, searched three databases, used an extensive snowball method and we did not apply any methodological criteria in the selection.

Regarding sports, exposure to volleyball [10] and basketball [22-24], with training and playing hours of at least 12 hours per week and/or in combination with weight training of at least 5 hours per week [7,23] and/or with playing or training on a hard surface [7] seems associated with an increased prevalence of jumper's knee. These activities probably result in a high load on the patellar tendon, which might increase the risk of developing jumper's knee. Taking into account the younger age and higher fitness level of the elite athletes compared to the 'average worker', these results hamper generalisation of the work-relatedness of jumper's knee for the occupational setting. In occupations at risk, a basic requirement is probably that high patellar loads should be present. This might be the case in occupations in which physical training is a part of the job, such as in police-work or in fire-fighting. However, exposure in terms of training hours might be relatively low. Moreover, knee-straining activities such as the frequent climbing of stairs while handling loads and/or jumping of objects might be risk factors for window-cleaners, construction workers, and truck-drivers that handle packaged goods [15].

Finally, there was a lack of consensus about case definitions for jumper's knee, making it hard to compare the results of different studies. Studies should use unequivocal case-definitions of jumper's knee and use similar diagnostic methods so that the results can be compared.

## Conclusion

Risk factors in sports that are associated with the onset or worsening of jumper's knee are exposure in volleyball and basketball, in combination with training and playing hours of at least 12 hours per week and/or in combination with weight training of at least 5 hours per week, and/or with playing or training on a hard surface. We did not find any specific occupational risk factor.

## Competing interests

The authors declare that they have no competing interests.

## Authors' contributions

IT, PK and MF conceived and designed the study. IT drafted the manuscript. PK, CH and MF obtained funding for this study. All authors read, made critical revisions and approved the final manuscript.
